# Aqueous extract of *Descuraniae* Semen attenuates lipopolysaccharide‐induced inflammation by inhibiting ER stress and WNK4‐SPAK‐NKCC1 pathway

**DOI:** 10.1111/jcmm.18589

**Published:** 2024-08-12

**Authors:** Po‐Chun Hsieh, Kun‐Lun Huang, Chung‐Kan Peng, Yao‐Kuang Wu, Guan‐Ting Liu, Chan‐Yen Kuo, Ming‐Chieh Wang, Chou‐Chin Lan

**Affiliations:** ^1^ Department of Chinese Medicine, Taipei Tzu Chi Hospital Buddhist Tzu Chi Medical Foundation New Taipei City Taiwan; ^2^ Department of Chinese Medicine National Yang Ming Chiao Tung University Taipei Taiwan; ^3^ Institute of Aerospace and Undersea Medicine National Defense Medical Center Taipei Taiwan; ^4^ Division of Pulmonary and Critical Care, Department of Internal Medicine Tri‐Service General Hospital Taipei Taiwan; ^5^ Department of Internal Medicine, Tri‐Service General Hospital National Defense Medical Center Taipei Taiwan; ^6^ Division of Pulmonary Medicine, Taipei Tzu Chi Hospital Buddhist Tzu Chi Medical Foundation New Taipei City Taiwan; ^7^ School of Medicine Tzu Chi University Hualien Taiwan; ^8^ Department of Research, Taipei Tzu Chi Hospital Buddhist Tzu Chi Medical Foundation New Taipei City Taiwan; ^9^ Department of Pharmacy, Taipei Tzu Chi Hospital Buddhist Tzu Chi Medical Foundation New Taipei City Taiwan

**Keywords:** acute lung injury, *Descuraniae* Semen, sepsis, sodium‐potassium‐chloride co‐transporter 1

## Abstract

Sepsis causes systemic inflammatory responses and acute lung injury (ALI). Despite modern treatments, sepsis‐related ALI mortality remains high. Aqueous extract of *Descuraniae* Semen (AEDS) exerts anti‐endoplasmic reticulum (ER) stress, antioxidant and anti‐inflammatory effects. AEDS alleviates inflammation and oedema in ALI. Sodium‐potassium‐chloride co‐transporter isoform 1 (NKCC1) is essential for regulating alveolar fluid and is important in ALI. The NKCC1 activity is regulated by upstream with‐no‐lysine kinase‐4 (WNK4) and STE20/SPS1‐related proline/alanine‐rich kinase (SPAK). This study aimed to investigate the effects of AEDS on lipopolysaccharide (LPS)‐induced ALI model in A549 cells, considering the regulation of ER stress, WNK4‐SPAK‐NKCC1 cascades, inflammation and apoptosis. Cell viability was investigated by the CCK‐8 assay. The expressions of the proteins were assessed by immunoblotting analysis assays. The levels of pro‐inflammatory cytokines were determined by ELISA. The expression of cytoplasmic Ca^2+^ in A549 cells was determined using Fluo‐4 AM. AEDS attenuates LPS‐induced inflammation, which is associated with increased pro‐inflammatory cytokine expression and activation of the WNK4‐SPAK‐NKCC1 pathway. AEDS inhibits the WNK4‐SPAK‐NKCC1 pathway by regulating of Bcl‐2, IP3R and intracellular Ca^2+^. WNK4 expression levels are significantly higher in the WNK4‐overexpressed transfected A549 cells and significantly decrease after AEDS treatment. AEDS attenuates LPS‐induced inflammation by inhibiting the WNK4‐SPAK‐NKCC1 cascade. Therefore, AEDS is regarded as a potential therapeutic agent for ALI.

## 1 INTRODUCTION

Sepsis, the leading cause of death worldwide, is believed to cause systemic inflammatory responses with increased levels of pro‐inflammatory cytokines, such as interleukin (IL)‐1β, IL‐6, IL‐8 and tumour necrosis factor α (TNF‐α), causing diffuse alveolar damage.^1^ The lung is a fragile organ with 40% of patients having acute lung injury (ALI) attributable to sepsis in response to systemic inflammatory responses.^2^ Sepsis‐related ALI is characterized by inflammatory cell infiltration, alveolar epithelium damage, pulmonary oedema and impaired gas exchange.^3^ This leads to the clinical presentation of refractory hypoxaemia and respiratory failure that require mechanical ventilation.^1^, ^4^ Despite modern treatment, sepsis‐related ALI and mortality remain high. Therefore, developing potentially effective treatments for ALI requires understanding its pathobiology.

*Descuraniae* Semen (DS) is derived from *Descurainia sophia* L. Webb ex Prantl.^5^ It has traditionally been used in medicine globally. Aqueous extract of DS (AEDS) is an aqueous extract of the seed of *D. sophia*.^5^ In clinical practice, AEDS has been used to treat many pulmonary diseases, such as cough, asthma and pulmonary oedema for a long time.^6^ Clinical studies of DS have not shown significant adverse effects, indicating that it is safe and effective.^6^ AEDS is known to exert antioxidative and anti‐inflammatory effects.^7^, ^8^ AEDS combined with current conventional treatment strategies for ALI improved pulmonary vascular permeability, decreased lung water, improved oxygenation and reduced mechanical ventilation days, and decreased mortality rate significantly better than current conventional treatment strategies alone.^9^, ^10^ It is reported AEDS decreased pulmonary oedema and inflammation in rats with ALI that was induced by LPS.^11^ These studies showed that AEDS might be an effective treatment for ALI. However, the molecular mechanism of AEDS in ALI remains to be understood.

Previous studies have demonstrated the regulatory roles of endoplasmic reticulum (ER) stress and unfolded protein response (UPR) in LPS‐induced ALI. Endo et al. revealed that administration of LPS elevated the expression of TNF‐α and IL‐1β, which increased ER stress and activated the UPR pathway, including BiP, CHOP and caspase‐11 induction in C57BL/6 mice and mice peritoneal macrophages.^12^ Li et al. reported that in A549 cells, LPS‐activated ER stress and autophagy, which was attenuated by ER stress inhibitor 4‐phenylbutyrate (4‐PBA). This study reported that LPS dominantly activated the PERK/p‐eIF2α/ATF4 pathway in A549 cells.^13^ Kim et al. reported that 4‐PBA attenuated LPS‐induced lung inflammation and decreased the LPS‐induced elevated expressions of BiP, CHOP, XBP1, ATF6, ATF4 and p‐eIF2α in the LPS‐induced ALI mouse model.^14^ In our previous study, we reported that AEDS enhances adaptive ability against ER stress by regulating proteasomal degradation.^15^ These studies demonstrated that ER stress is one important regulator in LPS‐induced ALI.

There are two isoforms of sodium‐potassium‐chloride co‐transporters (NKCC1 and NKCC2). Only NKCC1 is present in lung tissues.^16^ NKCC1 localized at the basolateral surface of alveolar cells is function to drive water transport through the back transport of sodium (Na^+^) and chloride (Cl^−^) to the alveolar space. It is known that NKCC1 is essential for regulating alveolar fluid and is important in ALI.^17^ With‐no‐lysine kinase‐4 (WNK4) and STE20/SPS1‐related proline/alanine‐rich kinase (SPAK) are related in the signalling cascade that regulates NKCC1 activity. High expression of WNK4‐SPAK‐NKCC1 results in alveolar oedema and inflammation.^16^ Therefore, the manipulation of NKCC1 expression is a strategy to treat ALI. AEDS has diuretic effects with the inhibition of reabsorption of water, Na^+^ and chloride Cl^−^ in renal tubules.^6^ However, the mechanism underlying the diuretic effects of AEDS is unknown. Loop diuretics are common and act by inhibiting NKCC channels in Henle's loop of the kidney.^18^ Since AEDS has diuretic effects, whether AEDS regulates NKCC1 in lung epithelial cells remains to be seen.

AEDS has anti‐inflammatory effects; however, the detailed mechanisms underlying its therapeutic effects remain unclear. The aim of this study was to investigate how AEDS regulates WNK4‐SPAK‐NKCC1 and inflammation in LPS‐induced ALI in A549 cells. Clinical treatment for patients with sepsis‐related ALI can be improved with increased knowledge about AEDS.

## 2 MATERIALS AND METHODS

### 2.1 Reagents

The concentrated aqueous extract power of DS (Ting Li Zi, K850, #421312802) was provided by Ko Da Pharmaceutical Co., Ltd. (Taoyuan, Taiwan). One gram of the concentrated AEDS was derived from the 5 g of DS. LPS was purchased from Sigma‐Aldrich (St. Louis, MO, USA).

### 2.2 Experimental protocol

American Type Culture Collection (Manassas, VA, USA) provided us with the human lung epithelial cell line (A549). At 37°C, cells were cultured in 5% CO_2_ in Gibco BRL Life Technologies' Dulbecco's modified Eagle's medium (DMEM; Gibco BRL Life Technologies, Grand Island, NY, USA), supplemented with 10% fetal bovine serum (Gibco BRL Life Technologies, Grand Island, NY, USA) and 100 units/mL penicillin/streptomycin (100 units/mL). For 24 h, 1 × 10^6^ cells/well of A549 cells were plated in six‐well plates. The cells were harvested and extracted.

There were five groups in this experiment: control, LPS, AEDS, AEDS pre‐treatment (preAEDS) and AEDS treatment (postAEDS) (Figure S1). The control group was A549 cells cultured in DMEM. The LPS group was A549 cells exposed to LPS (Sigma‐Aldrich, St. Louis, MO, USA). LPS was dissolved in DMEM to a concentration of 50 μg/mL. The AEDS group was A549 cells exposed to AEDS. The AEDS concentrates (Taoyuan, Taiwan, Ko Da Pharmaceutical Co.) were dissolved in DMEM at a final concentration of 200 g/mL. The preAEDS group was pre‐treated with AEDS followed by LPS. The postAEDS group consisted of A549 cells exposed to LPS and treated with AEDS.

### 2.3 Cell viability assay

Cell Counting Kit‐8 (CCK8, Dojindo, Kumamoto, Japan) was used to detect cell viability in the sample.^19^ It was used in conjunction with instructions provided by the manufacturer for detecting cell viability in the sample. Overnight incubation was performed with 2 × 10^4^ cells per well in 96‐well plates. Each well of the plate was then filled with 10 μL of CCK‐8 solution and exposed to AEDS and LPS concentrations indicated above for 2 h. The absorbance at 450 nm was measured with an Infinite 200 PRO microplate reader (Tecan, Männedorf, Switzerland).

### 2.4 Immunoblotting analysis

To collect and lyse the treated cells, we used a cold radioimmunoprecipitation assay buffer containing protein inhibitors. SDS–polyacrylamide gels were used to resolve proteins, and PVDF membranes were used to transfer them. The immunoblotting was conducted using primary antibodies and secondary antibodies conjugated with horseradish peroxidase. Peroxidase activity was measured by enhanced chemiluminescence. Analysing the intensities of reactive bands was performed with Bio‐Rad ChemiDoc XRS+ (Bio‐Rad, Hercules, CA, USA).

### 2.5 Expression of pro‐inflammatory cytokines

A549 cells (1 × 10^6^) in six‐well plates were incubated at 37°C for 24 h in a humidified incubator. Each of the five experimental groups cultured cells in the same way. After the experiment, the medium was removed from the well and stored at −80°C until the assay was conducted. According to the manufacturer's instructions, commercial enzyme‐linked immunosorbent assay (ELISA) kits (ABclonal Inc., Woburn, MA, USA) were used to determine the levels of cytokines in the medium.

### 2.6 Measurement of Ca^2+^ concentrations

Fluo‐4 AM (Thermo, Waltham, MA, USA) was used to monitor free [Ca^2+^] within the cell. The expression of cytoplasmic Ca^2+^ in A549 cells was determined using Fluo‐4 AM. For 1 h, A549 cells in medium (5 mM Fluo‐4 AM, 37°C) were incubated with Fluo‐4 AM in poly L‐lysine‐coated 96‐well black‐walled plates. We washed and treated the cells with the appropriate medium for each group. Using an Infinite 200 PRO microplate reader (Tecan, Männedorf, Switzerland), we measured fluorescence at intervals of 10 s (excitation 488 nm, emission 525 nm).

### 2.7 Intracellular Ca^2+^ chelator BAPTA, AM

BAPTA, AM (Thermo, Waltham, MA, USA) is a chelator that is highly selective for Ca^2+^. BAPTA, AM effectively blocked intracellular Ca^2+^ levels. We treated the cells with AEDS for 1 h, LPS for 16 h or Ca^2+^ chelator BAPTA, AM (10 μM) alone for 2 h or pretreated with BAPTA and AM, followed by AEDS. We collected and lysed the cells in cold RIPA buffer with protein inhibitors and stored them at 80°C until they were used.

### 2.8 Ionomycin

Ionomycin (MedChemExpress, NJ, USA) is a Ca^2+^ ionophore that elevates intracellular Ca^2+^ concentrations. We treated the cells with AEDS (200 μg/mL) or ionomycin (10 μg/mL) alone for 1 h or pretreated with ionomycin, followed by AEDS. We collected and lysed the cells in cold RIPA buffer with protein inhibitors and stored them at 80°C until they were used.

### 2.9 Intracellular Ca^2+^ chelator BAPTA, AM

BAPTA, AM (Thermo, Waltham, MA, USA) is a chelator that is highly selective for Ca^2+^. BAPTA, AM effectively blocked intracellular Ca^2+^ levels. We treated the cells with AEDS for 1 h, LPS for 16 h, Ca^2+^ chelator BAPTA, AM (10 μM) alone for 2 h or pretreated with BAPTA and AM, followed by AEDS. We collected and lysed the cells in cold RIPA buffer with protein inhibitors and stored them at 80°C until they were used.

### 2.10 Plasmid constructs

WNK4 overexpression plasmid constructs were commissioned from BIOTOOLS Co. (New Taipei City, Taiwan). Human WNK4 [NM_001321299.2] were cloned into pLV[Exp]‐Puro‐CMV‐vector (vector ID: VB240419‐1149pcy).

### 2.11 Generation of WNK4 overexpression stable cell lines

A549 cells (1 × 10^6^ cells) were cultured in a plate and incubated at 37°C in a humidified incubator. Afterward, pLV[Exp]‐Puro‐CMV or pLV[Exp]‐Puro‐CMV‐hWNK4 was transfected into cells by TOOLS smoothFect transfection reagent (BIOTOOLS Co., New Taipei City, Taiwan). At 24 h after transfection, stable transfectants were selected with puromycin, and the selection medium thereafter was replaced every other day. After 2 weeks of selection, resistant clones were established.

### 2.12 Statistical analysis

The statistical analysis was conducted using GraphPad Prism 10 (Version 10.2.3 (347); GraphPad Software, San Diego, CA, USA). The mean and standard deviation of the experiment are presented. To compare parameters between groups, we used one‐way ANOVA and Tukey's multiple comparison test. Statistical significance is determined by a *p*‐value less than 0.05.

## 3 RESULTS

### 3.1 AEDS inhibits LPS‐induced ER stress in A549 cells

Immunoblotting analysis results of BiP and UPR cascades (pIRE1α, pJNK, Bcl‐2) in the experimental groups are presented in Figure S2. The expression levels of BiP, pIRE1α and pJNK are significantly higher in the LPS group compared with the control group (Figure S2B–D). The expression levels of BiP, pIRE1α and pJNK are significantly lower in the preAEDS and postAEDS groups compared with the LPS group (Figure S2B–D). The expression level of Bcl‐2 is significantly lower in the LPS group compared with the control group, but significantly higher in the preAEDS group compared with the LPS group (Figure S2E). Based on these results, AEDS inhibited LPS‐induced ER stress in A549 cells.

### 3.2 AEDS inhibits LPS‐induced IP3Rs activation in A549 cells

According to the results, we performed an immunoblotting analysis of the expression level of inositol 1,4,5‐trisphosphate receptor (IP3R), inhibited by Bcl‐2 and predominantly responsible for transporting Ca^2+^ from the ER to the cytoplasm and mitochondria.^20^ Immunoblotting analysis result of the expression of IP3R in the experimental groups is presented in Figure S2F. The expression levels of IP3R are significantly higher in the LPS and AEDS groups compared with the control group. However, the expression level of IP3R is significantly higher in the LPS group compared with the AEDS group. Compared with the LPS group, IP3R levels are significantly lower in the preAEDS and postAEDS groups.

### 3.3 AEDS inhibits LPS‐induced elevation of intracellular calcium (Ca^2+^) levels in A549 cells

**FIGURE 1:**
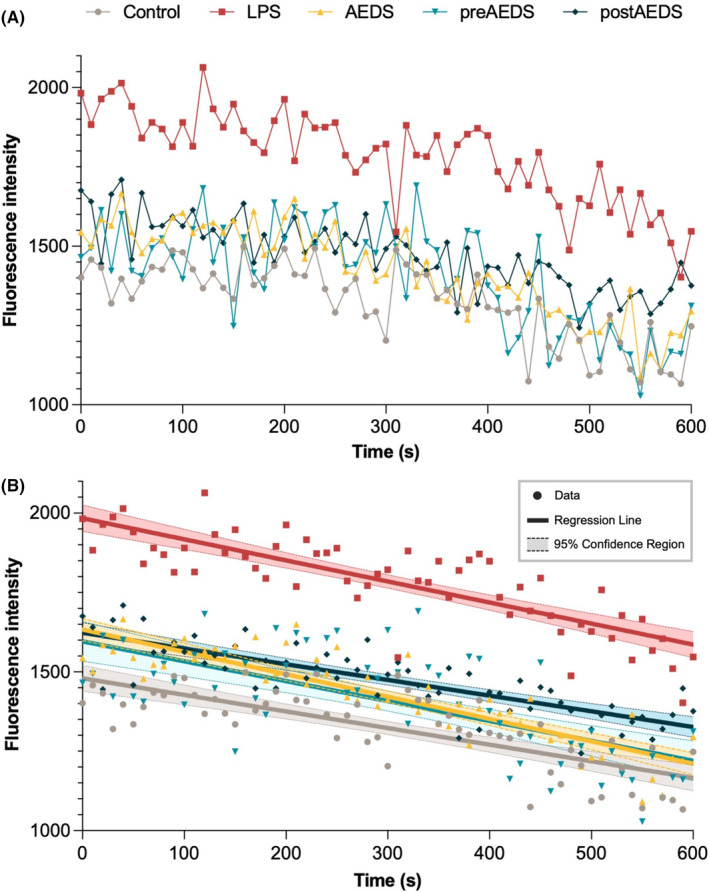
AEDS inhibits LPS‐induced intracellular Ca^2+^ levels elevation in A549 cells. (A) Fluo‐4 AM fluorescence intensity and (B) Linear regression of the fluorescence intensity data in the five experimental groups. Fluorescent signals (excitation 488 nm, emission 525 nm) were recorded at intervals of 10 s. The intensity of the Fluo‐4 AM fluorescent signals represent the relative level of intracellular calcium. Three duplicates of the experiment were conducted.

We also investigated the intensity of Fluo‐4 AM fluorescent signals, which represent relative levels of intracellular Ca^2+^ (Figure 1A). The intercept test of the linear regression results showed significantly higher intracellular Ca^2+^ levels in the LPS group than in other groups (Figure 1B). The intercept test of the linear regression results also showed significantly higher intracellular Ca^2+^ levels in the AEDS (*p* < 0.001), preAEDS (*p* = 0.002) and postAEDS (*p* < 0.001) groups than in the control and LPS groups (Figure 1B).

### 3.4 LPS activates WNK4 by elevating intracellular Ca^2+^ levels

**FIGURE 2:**
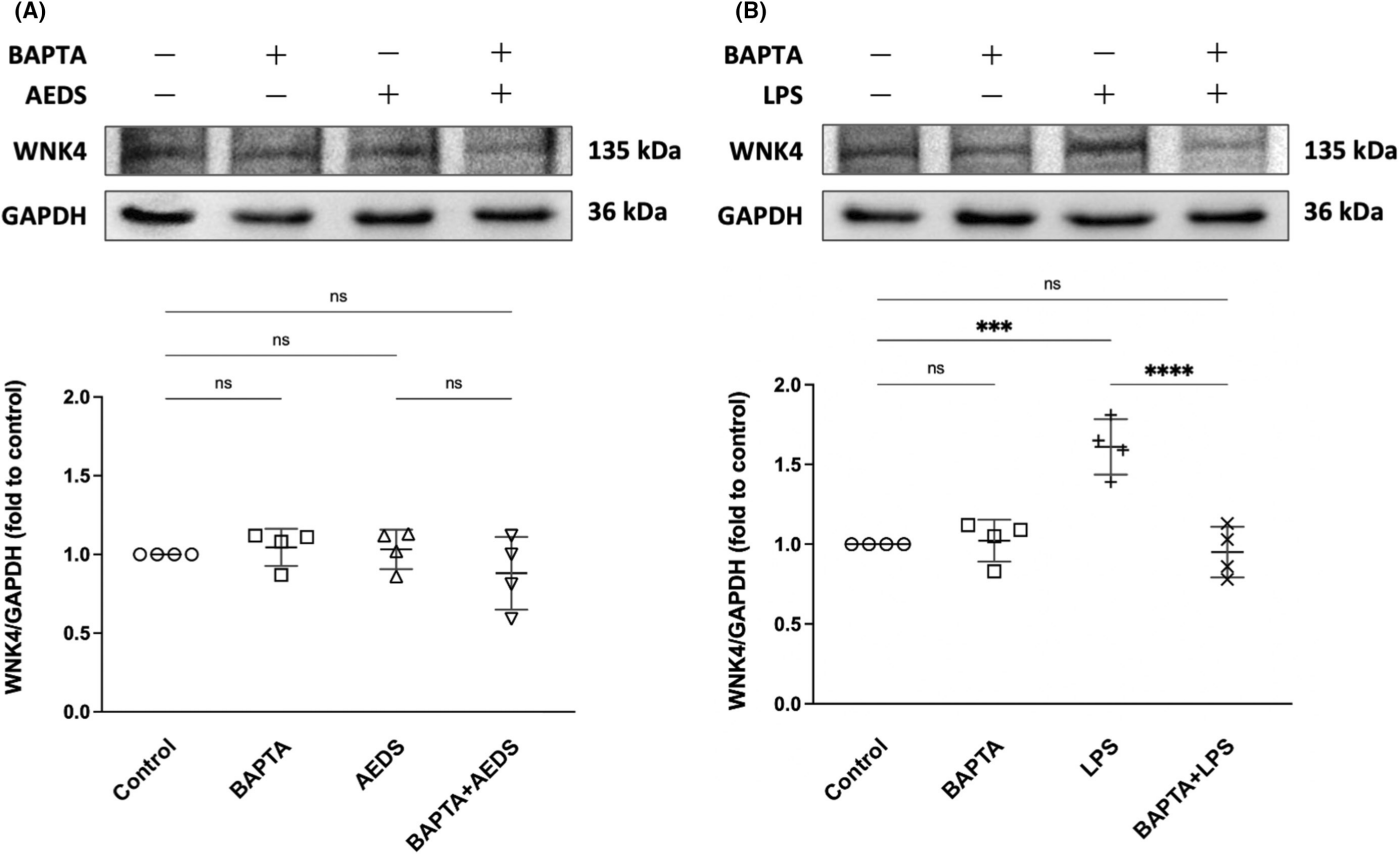
LPS activates WNK4 by elevating intracellular Ca^2+^ levels. Immunoblotting results of (A) BAPTA (10 μM, 2 h) and AEDS (200 μg/mL, 1 h), and (B) BAPTA (10 μM, 2 h) and LPS (50 μg/mL, 16 h) on WNK4 expression. All data are expressed as mean ± SD. Four duplicates of the experiment were conducted. ***p < 0.001, ****p < 0.0001.

We performed immunoblotting analysis on the expression levels of WNK4 induced by AEDS (200 μg/mL, 1 h), LPS (50 μg/mL, 16 h) or BAPTA (10 μM, 2 h) in A549 cells (Figure 2). WNK4 expression levels after AEDS, BAPTA or BAPTA+AEDS induction were not significantly different (Figure 2A). When compared with control conditions, WNK4 expression is significantly higher after LPS induction (Figure 2B). The expression level of WNK4 is significantly lower after BAPTA+LPS induction compared with LPS induction and showed no difference compared with those in the control condition (Figure 2B). The results indicate that LPS increase the expression level of WNK4 by elevating intracellular Ca^2+^ levels, whereas AEDS had no prominent effects on the expression of WNK4.

### 3.5 AEDS inhibits ionomycin‐induced WNK4 activation in A549 cells

**FIGURE 3:**
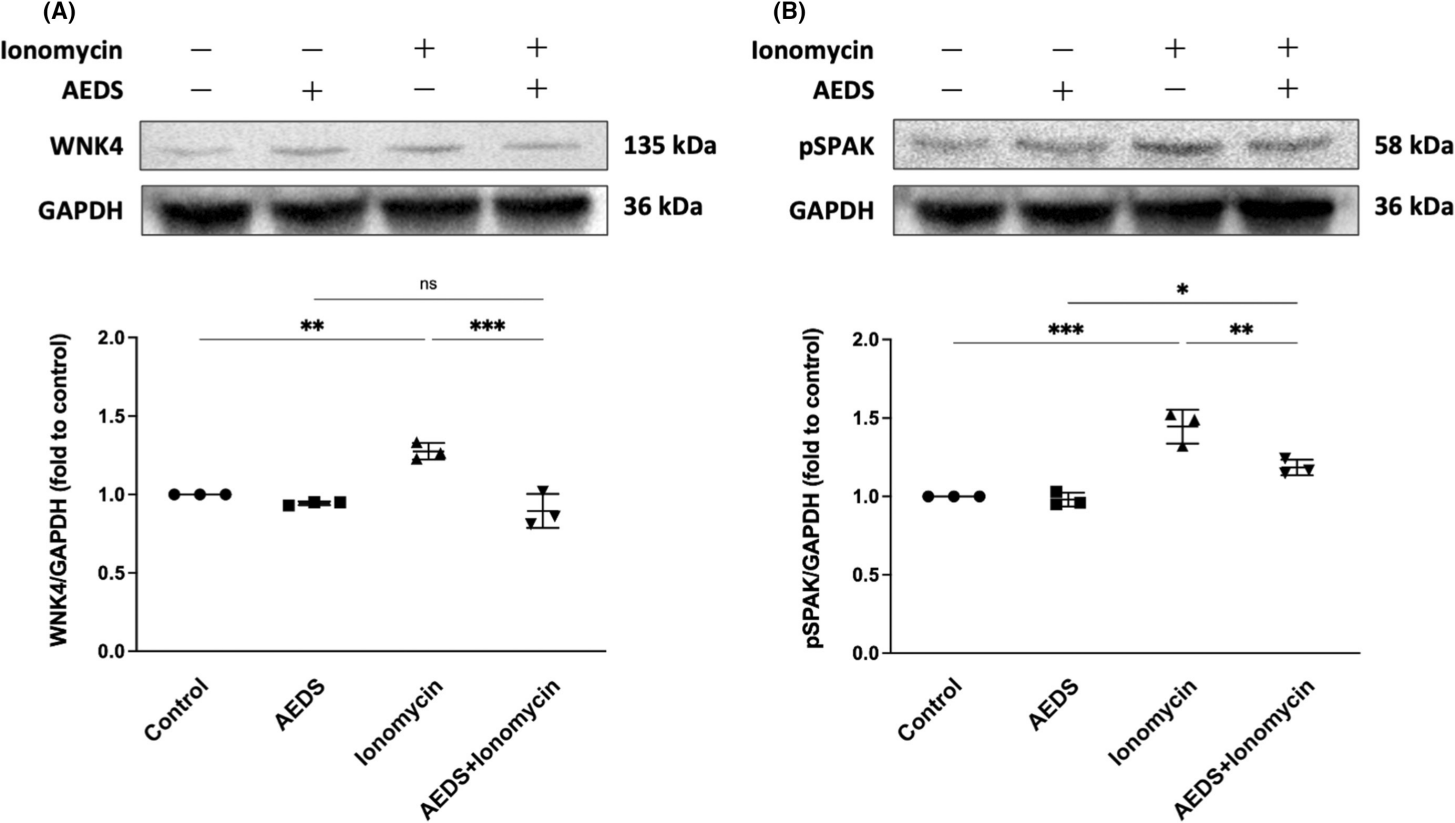
AEDS inhibits ionomycin‐induced WNK4 activation in A549 cells. Immunoblotting results show the effects of AEDS at 200 μg/mL for 1 hour (A) and ionomycin at 50 μg/mL for 16 hours (B) on WNK4 and pSPAK expression

We performed immunoblotting analysis on the expression levels of WNK4 and pSPAK induced by AEDS (200 μg/mL, 1 h) or ionomycin (10 μg/mL, 1 h) in A549 cells. The expression levels of WNK4 and pSPAK are significantly higher after ionomycin induction compared with those in the control conditions (Figure 3A,B). The expression level of WNK4 and pSPAK is significantly lower after AEDS+ionomycin induction compared with ionomycin induction (Figure 3A,B). The results indicate that AEDS inhibits intracellular calcium level related WNK4‐pSPAK pathway activation.

**FIGURE 4:**
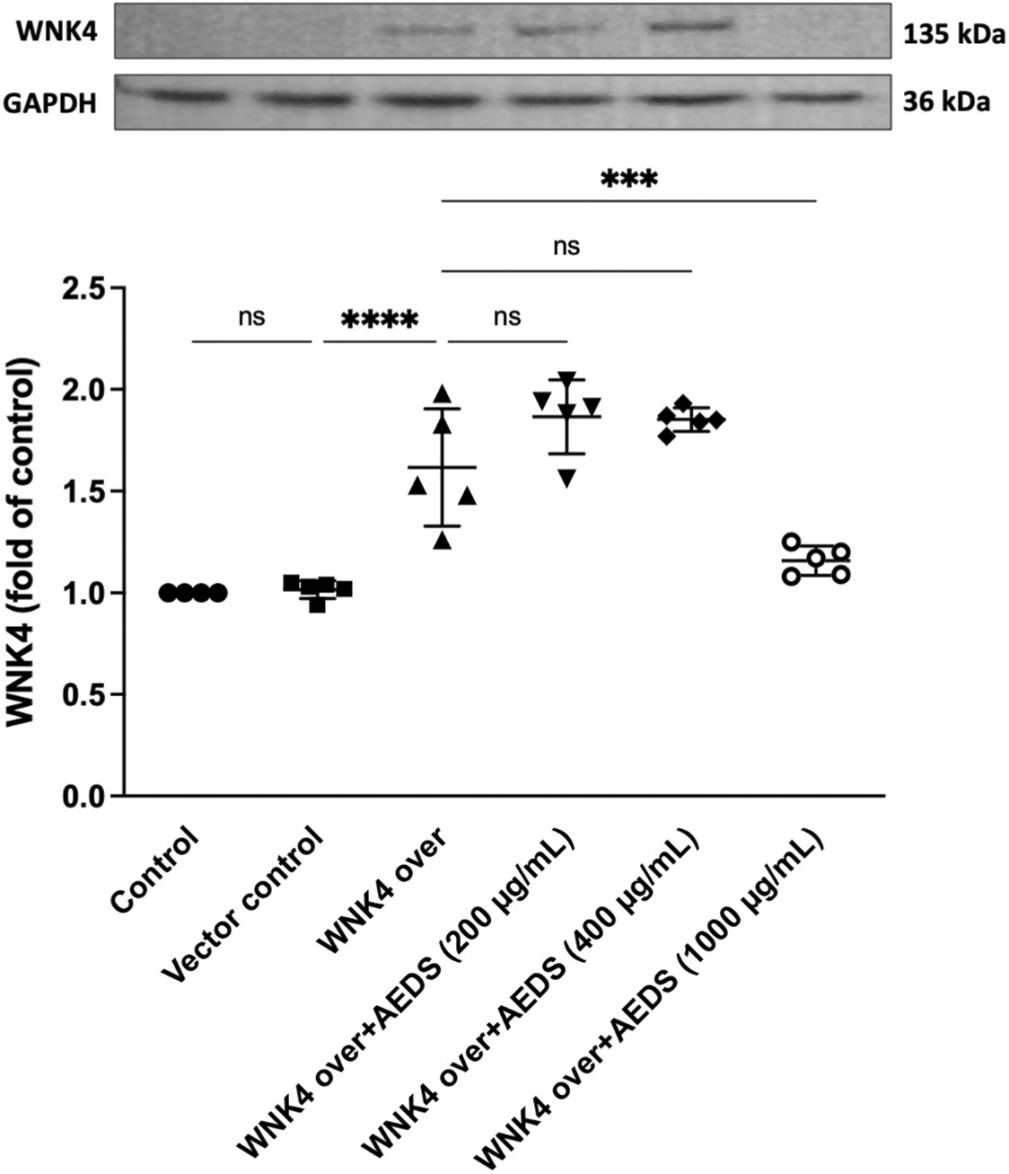
AEDS inhibits WNK4 expression in WNK4‐overexpressed A549 cells. Immunoblotting results of WNK4 expression levels in A549 cells, vector‐transfected A549 cells, WNK4‐overexpressed A549 cells, AEDS‐treated (200, 400 or 1000 μg/mL, 1 h) WNK4‐overexpressed A549 cells. WNK4 expression levels are significantly higher in the WNK4‐overexpression group than in the vector control group. Treatment with AEDS (1000 μg/mL, 1 h) significantly decreased WNK4 expression compared to the WNK4‐overexpression group. All data are expressed as mean ± SD. Five duplicates of the experiment were conducted. ***p < 0.001, ****p < 0.0001.

### 3.6 AEDS inhibits WNK4 expression in WNK4‐overexpressed A549 cells

We performed an immunoblotting analysis to assess the expression levels of WNK4 in various A549 cell groups. These groups included untreated A549 cells, vector‐control A549 cells, WNK4‐overexpressing A549 cells, and WNK4‐overexpressing A549 cells treated with AEDS at concentrations of 200, 400, or 1000 μg/mL for 1 hour. The expression levels of WNK4 are significantly higher in the WNK4‐overexpression group compared with the vector control group (Figure 4). The expression levels of WNK4 significantly decreased after treated by AEDS (1000 μg/mL, 1 h) compared with the WNK4‐overexpression group. The results indicate that AEDS inhibits WNK4 expression in WNK4‐overexpressed A549 cells.

### 3.7 AEDS inhibits LPS‐induced WNK4‐SPAK‐NKCC1 signalling activation in A549 cells

**FIGURE 5:**
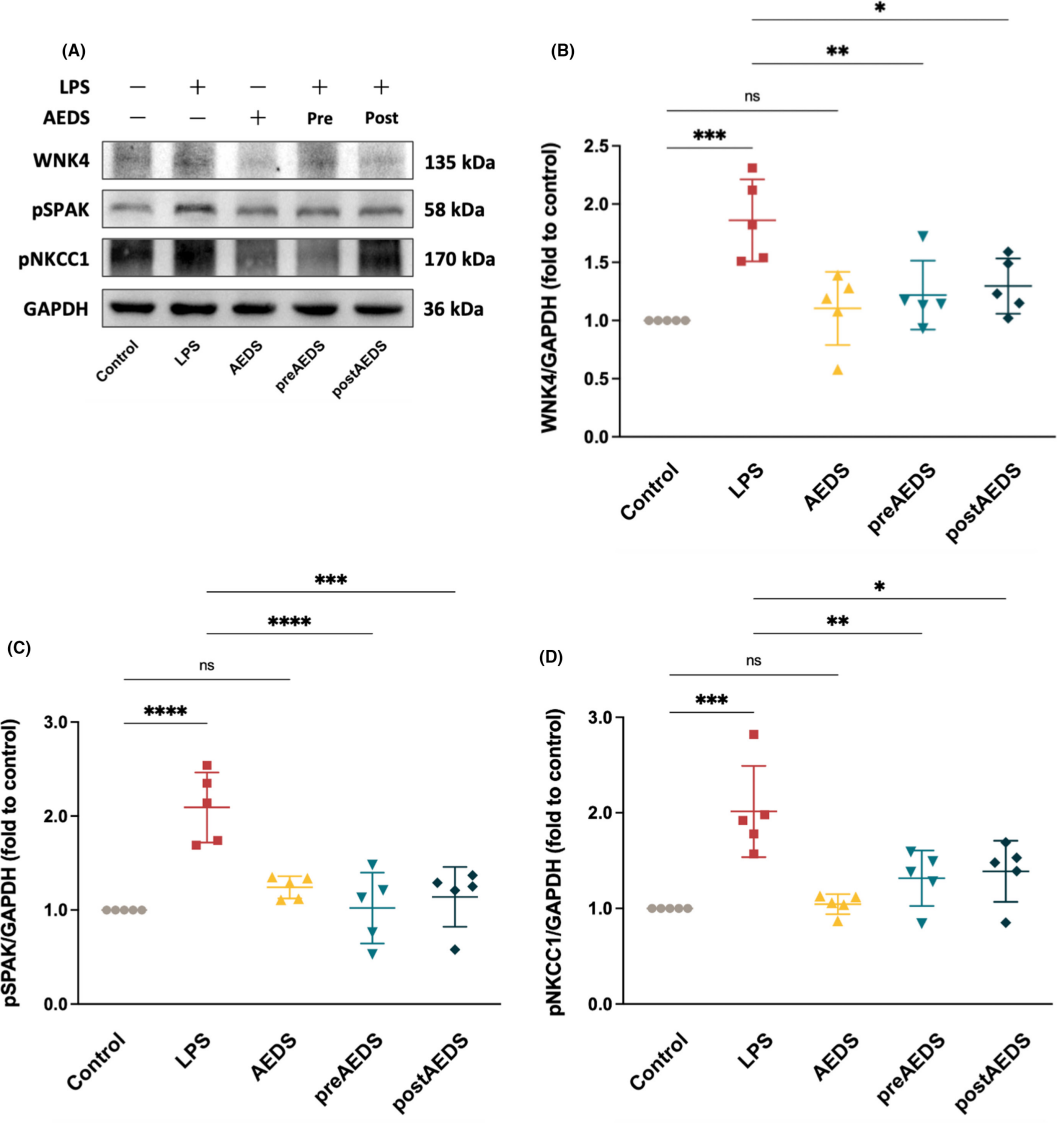
AEDS inhibits LPS‐induced WNK4‐SPAK‐NKCC1 signalling activation in A549 cells. (A) Immunoblotting results for WNK4, pSPAK and pNKCC1. Quantitative western blot results for WNK4 (B), pSPAK (C) and pNKCC1 (D) expression. All data are expressed as mean ± SD. Four duplicates of the experiment were conducted. *p < 0.05, **p < 0.01, ***p < 0.001, ****p < 0.0001.

Immunoblotting analysis results for WNK4, phosphorylated SPAK (pSPAK) and phosphorylated NKCC1 (pNKCC1) are presented in Figure 5. The expression levels of WNK4, pSPAK and pNKCC1 are significantly higher in the LPS group compared with the control group (Figure 5B–D). The levels of WNK4, pSPAK and pNKCC1 are significantly lower in the preAEDS and postAEDS groups compared with the LPS group (Figure 5B–D). Activation of WNK4‐SPAK‐NKCC1 signalling by LPS is inhibited by AEDS in A549 cells.

### 3.8 AEDS inhibits LPS‐induced elevated cytokine expression in A549 cells

**FIGURE 6:**
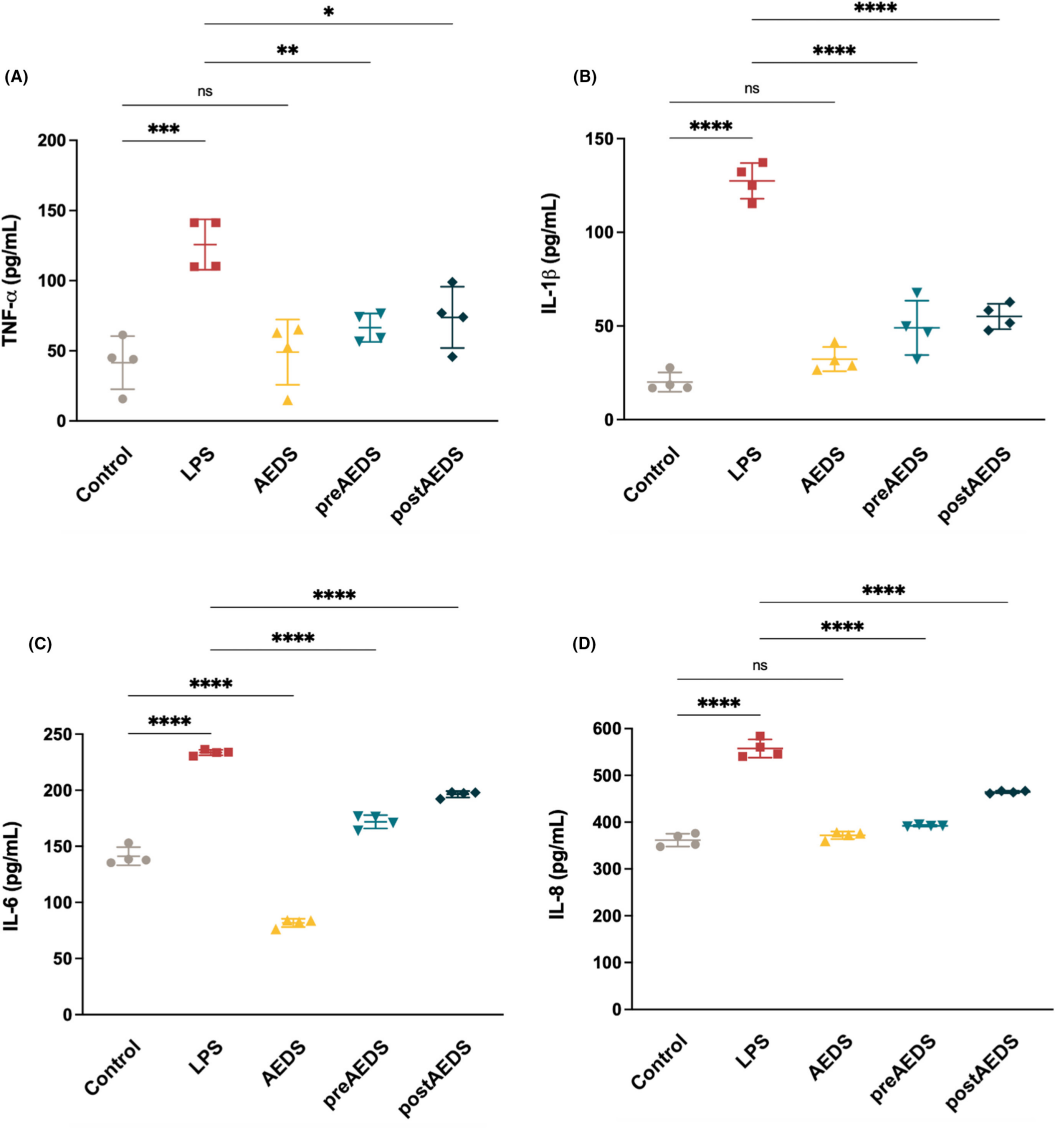
AEDS inhibits LPS‐induced increase in pro‐inflammatory cytokine levels. ELISA results of (A) TNF‐α, (B) IL‐1β, (C) IL‐6 and (D) IL‐8 levels in the culture medium. Four duplicates of the experiment were conducted. *p < 0.05, **p < 0.01, ***p < 0.001, ****p < 0.0001.

The ELISA results for the levels of TNF‐α, IL‐1β, IL‐6 and IL‐8 are presented in Figure 6. The levels of TNF‐α, IL‐1β, IL‐6 and IL‐8 in the LPS group were significantly higher than those in the control group (Figure 6A–D). TNF‐α, IL‐1β, IL‐6 and IL‐8 levels were significantly lower in the preAEDS and postAEDS groups compared with those in the LPS group (Figure 6A–D). These indicate that AEDS attenuated LPS‐induced inflammation in A549 cells.

## 4 DISCUSSION

**FIGURE 7:**
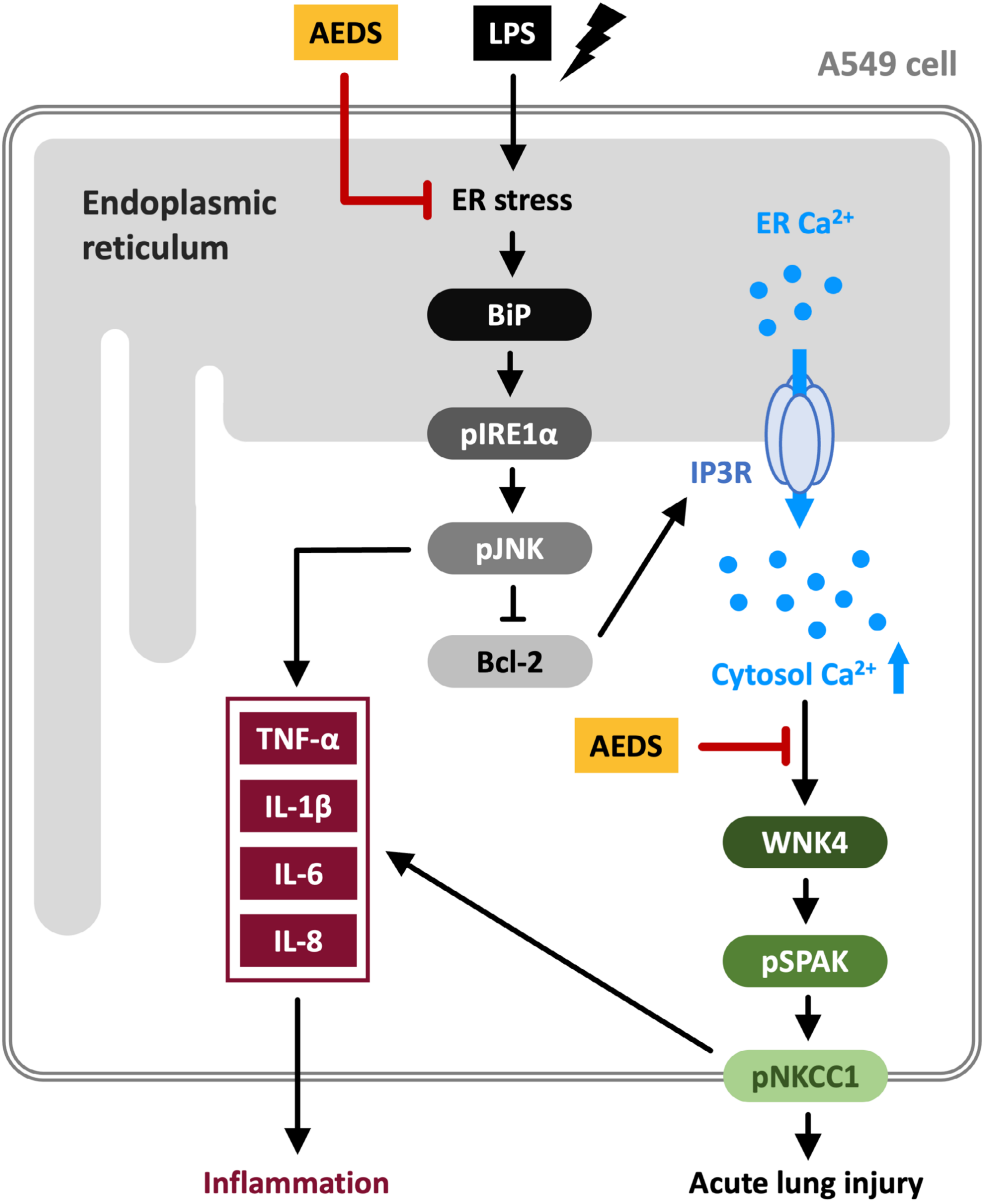
AEDS inhibits LPS‐induced ER stress, inflammation, IP3R activation, intracellular Ca^2+^ levels elevation and WNK4‐SPAK‐NKCC1 signalling activation in A549 cells. LPS induced ER stress with increased expression of BiP and pIRE1α and activated the downstream pathway of JNK and pro‐inflammatory cytokines. This process also activates IP3R and increases intracellular Ca^2+^ levels, further activating the WNK‐SPAK‐NKCC1 pathway. The activated WNK‐SPAK‐NKCC1 pathway also increases the levels of proinflammatory cytokines. Administration of AEDS inhibited LPS‐induced ER stress by decreasing the expression of BiP and pIRE1α, and the downstream pathways of JNK and pro‐inflammatory cytokines. This process also deactivates IP3R and decreases intracellular Ca^2+^ levels, which further deactivates the WNK‐SPAK‐NKCC1 pathway and pro‐inflammatory cytokines.

This study has revealed the novel finding that we first demonstrated that AEDS inhibits LPS‐induced ER stress and WNK4‐SPAK‐NKCC1 signal activation. LPS induced ER stress with increased expression of BiP and pIRE1α and further activated the downstream pathway of JNK and pro‐inflammatory cytokines. This process activates IP3Rs and increases intracellular Ca^2+^ levels, which further activates the WNK‐SPAK‐NKCC1 pathways. The activated WNK‐SPAK‐NKCC1 pathway increases the levels of proinflammatory cytokines. Administration of AEDS inhibited LPS‐induced ER stress by decreasing the expression of BiP and pIRE1α and the downstream pathways of JNK and pro‐inflammatory cytokines. This process also deactivated IP3Rs and decreased intracellular Ca^2+^ levels, which further deactivated the WNK‐SPAK‐NKCC1 pathway and pro‐inflammatory cytokines. AEDS may have therapeutic potential in treating ALI based on these results. The mechanisms underlying the effects of AEDS on LPS‐induced ER stress and WNK‐SPAK‐NKCC1 pathways are shown in Figure 7.

Previously, DS components were shown to have anti‐inflammatory effects in many experimental studies.^7^, ^21^ Lee et al. demonstrated that DS components inhibit nitric oxide production in LPS‐stimulated macrophages.^7^ Nevertheless, these studies have not examined how AEDS reduce inflammation. In ALI, ER stress plays a crucial role in causing inflammation of the lungs. The UPR and ER stress play a role in ALI.^22^ Our previous studies showed that AEDS enhances adaptive ability against ER stress through the regulation of proteasome degradation.^15^ AEDS attenuates LPS‐triggered inflammation and apoptosis through modulation of proteasomal degradation, facilitation of IRE1α‐mediated adaptive UPR and suppression of IRE1α‐mediated apoptotic UPR.^15^

In ALI, the WNK4‐SPAK‐NKCC1 pathway contributes to lung inflammation and oedema. An increase in NKCC1 expression leads to an increase in alveolar fluid secretion and an impairment in alveolar fluid clearance. Through JNK‐ and p38‐dependent pathways, pro‐inflammatory cytokines also enhance NKCC1 expression.^16^ Overexpression of NKCC1 is associated with an increase in cell volume, which may lead to cell swelling during inflammation. This swelling can result in the rupture of the cellular membrane and the subsequent release of damage‐associated molecular patterns and inflammatory mediators.^16^ In addition, NKCC1 is also known to increase the mitogen‐activated protein kinase signalling pathway.^23^ The activation of NKCC1 therefore leads to the release of pro‐inflammatory cytokines, thereby exacerbating the inflammatory response. The interaction between WNK4‐SPAK‐NKCC1, along with pro‐inflammatory cytokines, leads a vicious cycle.^16^ Animals expressing higher levels of NKCC1 have been shown to have higher levels of pulmonary oedema, microvascular permeability and pro‐inflammatory cytokines, as well as a higher level of neutrophil infiltration.^17^, ^24^ Conversely, animals treated with NKCC1 inhibitors or animals with lower levels of NKCC1 expression suffer less severe lung injury.^17^, ^24^ Therefore, by inhibiting the WNK4‐SPAK‐NKCC1 pathway, AEDS may exert anti‐inflammatory effects and decrease fluid retention in ALI.

Ca^2+^ homeostasis is essential for many intracellular processes, including recruitment, activation and expression of NKCC1.^25^ One previous study revealed the regulation of NKCC1 is Ca^2+^‐dependent and that Ca^2+^ signals promote the recruitment, activation and expression of the NKCC1.^25^ Most intracellular Ca^2+^ is released from the ER. IP3Rs are important transporters that regulate Ca^2+^ influx or efflux.^26^ Bcl‐2 suppresses the Ca^2+^ release from the ER by inhibiting IP3R.^20^, ^27^ During ER stress, pIRE1α phosphorylates JNK. pJNK then inhibits Bcl‐2, which simultaneously activates IP3R and promotes the Ca^2+^ release from the ER.^28^ The previous studies demonstrated ER stress‐induced phosphorylation of JNK suppresses Bcl‐2.^29^, ^30^ According to this study, LPS exposure decreased Bcl‐2 expression while IP3Rs and intracellular Ca^2+^ were simultaneously increased in A549 cells. An excess of intracellular Ca^2+^ plays a key role in activating NKCC1 as a secondary messenger.^25^ AEDS increased Bcl‐2 and decreased the expression of IP3Rs expression and intracellular Ca^2+^ after LPS exposure. Decreased intracellular Ca^2+^ levels result in less NKCC1 and inflammation. Therefore, AEDS decreased the expression of the WNK4‐SPAK‐NKCC1 pathway by increasing the expression of Bcl‐2, inhibiting the expression of IP3R and preventing the elevation of intracellular Ca^2+^ levels.

The immunoblotting results demonstrate that WNK4 expression levels are significantly elevated in WNK4‐overexpressed A549 cells compared to vector‐transfected A549 cells. Treatment with AEDS at concentrations of 1000 μg/mL led to a significant decrease in WNK4 expression in the overexpressed cells. These findings suggest that AEDS downregulates WNK4 expression in a dose‐dependent manner. In previous studies, high expression of WNK4‐SPAK‐NKCC1 has been associated with inflammation.^16^ Consistent with these findings, our current study also demonstrates that LPS induced the activation of WNK4‐SPAK‐NKCC1. We suggest that AEDS has a potential therapeutic role in LPS‐related inflammation. However, further clinical studies are warranted.

### 4.1 Limitations

Some limitations were present in this study. First, in AEDS, approximately 67 compounds were identified.^31^ Among these, the component responsible for anti‐inflammation, the component that inhibits NKCC1, or the interaction between the components are unknown. Although this remains unclear, this study provides AEDS as a possible treatment modality for the treatment of ALI, since AEDS is a natural ingredient that has been used for a long time and has minimal side effects.^6^ Second, this study was performed in cells. Using AEDS, we were able to attenuate LPS‐induced cell injury by decreasing ER stress and the WNK4‐SPAK‐NKCC1 pathway. In order to confirm the effects of AEDS on ER stress and the WNK4‐SPAK‐NKCC1 pathway in patients with ALI, further clinical studies are required. Third, experiments on the effects of AEDS were performed in the early stages of LPS‐induced cell injury. T It remains unclear what the effects of AEDS will be in the long term and further research is needed.

## 5 CONCLUSIONS

ER stress and activation of the WNK4‐SPAK‐NKCC1 pathway are essential for ALI development. The novel finding of this study is that AEDS inhibits ER stress and the WNK4‐SPAK‐NKCC1 pathway. The mechanism by which AEDS acts in the WNK4‐SPAK‐NKCC1 pathway is the regulation of Bcl‐2 and intracellular Ca^2+^. Therefore, AEDS may serve as a potential therapeutic agent for ALI treatment. AEDS is a widely used herbal medicine and we explored its therapeutic mechanism of action in LPS‐related inflammation. Understanding the therapeutic mechanism can help in the clinical use of AEDS.

## AUTHOR CONTRIBUTIONS

**Po‐Chun Hsieh:** Conceptualization (equal); data curation (equal); writing – original draft (equal). **Kun‐Lun Huang:** Conceptualization (equal); data curation (equal); supervision (equal). **Chung‐Kan Peng:** Data curation (equal); investigation (equal); methodology (equal). **Yao‐Kuang Wu:** Conceptualization (equal); data curation (equal); supervision (equal). **Guan‐Ting Liu:** Conceptualization (equal); data curation (equal); supervision (equal). **Chan‐Yen Kuo:** Data curation (equal); investigation (equal); methodology (equal). **Ming‐Chieh Wang:** Data curation (equal); investigation (equal); methodology (equal). **Chou‐Chin Lan:** Conceptualization (equal); data curation (equal); funding acquisition (equal); writing – original draft (equal).

## FUNDING INFORMATION

Taipei Tzu Chi Hospital, Buddhist Tzu Chi Medical Foundation, New Taipei City, Taiwan supported this research (TCRD‐TPE‐112‐01, TCRD‐TPE‐112‐10).

## CONFLICT OF INTEREST STATEMENT

The authors declare no potential conflicts of interest.

## Supporting information


**Figure S1.** Study program. There were five groups in this experiment: control, LPS, AEDS, AEDS pre‐treatment (preAEDS) and AEDS treatment (postAEDS).

**Figure S2.** AEDS inhibits LPS‐induced ER stress and IP3R activation in A549 cells. (A) Immunoblotting results of BiP, pIRE1α, pJNK, Bcl‐2 and IP3R. Quantitative immunoblotting results of BiP (B), pIRE1α (C), pJNK (D), Bcl‐2 (E) and IP3R (F) expression. All data are expressed as the mean ± SD. Four duplicates of the experiment were conducted. *p < 0.05, **p < 0.01, ***p < 0.001, **** *p* < 0.0001.

## Data Availability

The data that support the findings of this study are available from the corresponding author upon reasonable request.
