# Supplementary figures and images for: Aqueous extract of Descuraniae Semen attenuates lipopolysaccharide‐induced inflammation by inhibiting ER stress and WNK4‐SPAK‐NKCC1 pathway
Source: J Cell Mol Med. 2024 Aug 12;28(15):e18589. doi: 10.1111/jcmm.18589 (PMC11319090; doi:10.1111/jcmm.18589)

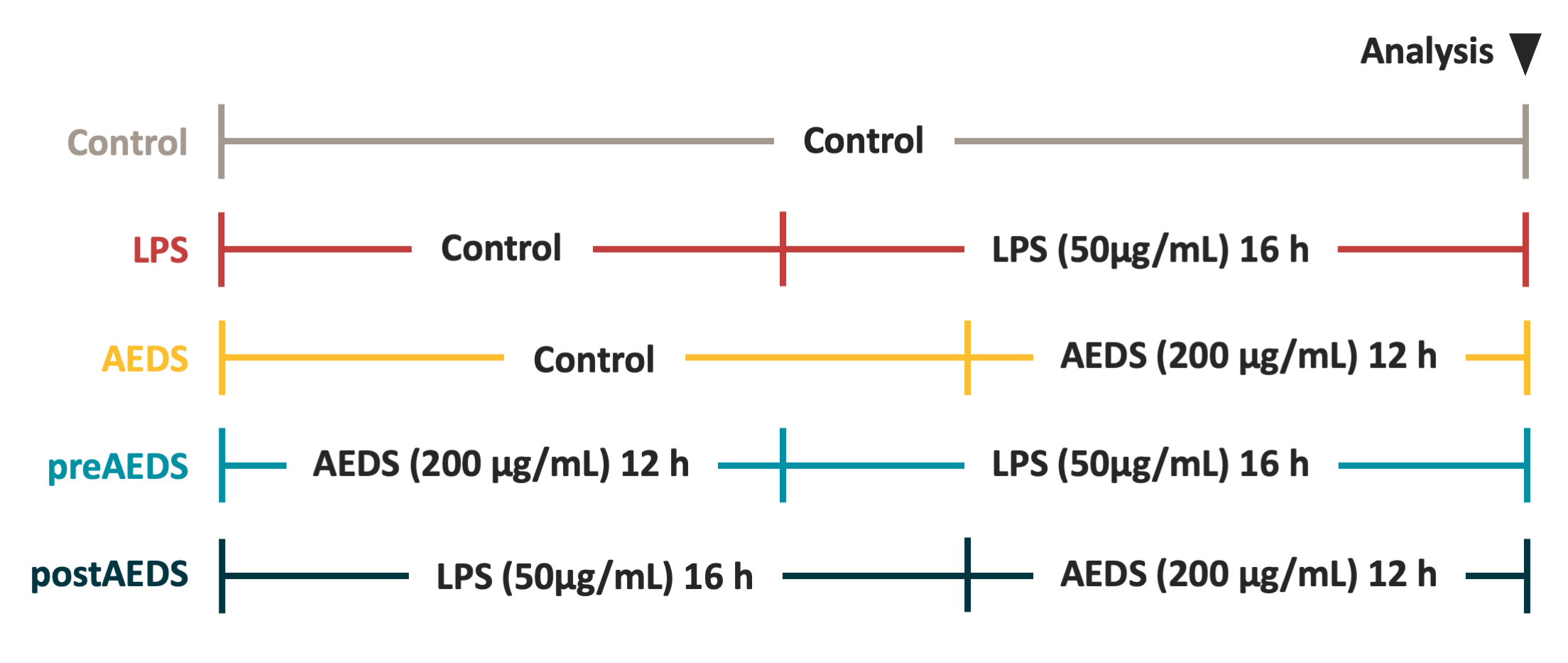

Supplement: Supplementary file 1 — Figure S1. Study program. There were five groups in this experiment: control, LPS, AEDS, AEDS pre‐treatment (preAEDS) and AEDS treatment (postAEDS). [file JCMM-28-e18589-s002.tiff]

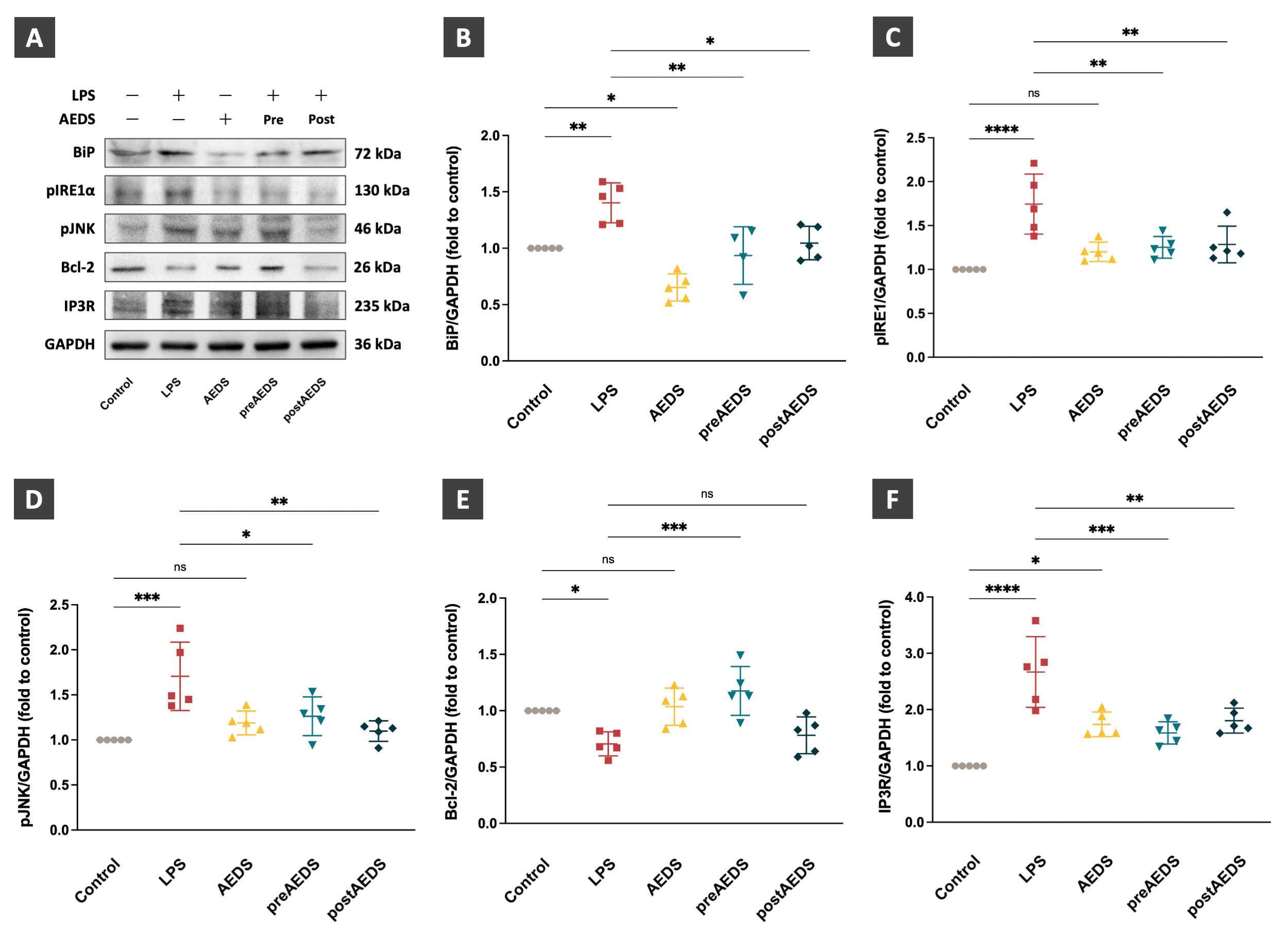

Supplement: Supplementary file 2 — Figure S2. AEDS inhibits LPS‐induced ER stress and IP3R activation in A549 cells. (A) Immunoblotting results of BiP, pIRE1α, pJNK, Bcl‐2 and IP3R. Quantitative immunoblotting results of BiP (B), pIRE1α (C), pJNK (D), Bcl‐2 (E) and IP3R (F) expression. All data are expressed as the mean ± SD. Four duplicates of the experiment were conducted. *p < 0.05, **p < 0.01, ***p < 0.001, **** p < 0.0001. [file JCMM-28-e18589-s001.tiff]
